# Cytokine-induced killer cells carrying recombinant oncolytic adenovirus expressing p21Ras scFv inhibited liver cancer

**DOI:** 10.7150/jca.51434

**Published:** 2021-03-10

**Authors:** Fang Dai, Peng-Bo Zhang, Qiang Feng, Xin-Yan Pan, Shu-Ling Song, Jing Cui, Ju-Lun Yang

**Affiliations:** 1 Graduate School, Kunming Medical University, Kunming, Yunnan, China.; 2 920 th Hospital of the Joint Logistics Support Force of PLA, Kunming, Yunnan, China.; 3 Medical School, Kunming University of Science and Technology, Kunming, Yunnan, China.

**Keywords:** oncolytic adenovirus, Ras, liver cancer, CIK, scFv

## Abstract

**Background:** Oncolytic adenovirus-mediated gene therapy is an emerging strategy for cancer treatment. However, oncolytic adenoviruses are mainly administered locally at tumor site. Intravenous administration of oncolytic adenovirus for cancer gene therapy is a problem that needs to be solved urgently.

**Methods:** We constructed recombinant oncolytic adenovirus KGHV500 carrying anti-p21Ras scFv and employed CIK cells to deliver KGHV500. TUNEL, wound healing, MTT, and Transwell invasion assays were used to determine the anti-tumor efficacy of KGHV500 on liver cancer cells* in vitro*. Nude mouse xenograft model was used to examine the anti-tumor efficacy of CIK cells combined with KGHV500 *in vivo*. Furthermore, KGHV500 accumulation in different organs was detected to assess the safety.

**Results:** KGHV500 inhibited the migration, proliferation, invasion, and induced the apoptosis of liver cancer cells. CIK cells carrying KGHV500 reached tumor site and exerted much better anti-tumor efficacy than CIK cells or KGHV500 alone in nude mouse xenograft model. Moreover, we detected KGHV500 and anti-p21Ras scFv in different organs of nude mice, with little effects on the organs.

**Conclusions:** We develop a novel strategy for the treatment of Ras-driven liver cancer by combining CIK cells with oncolytic adenovirus expressing anti-p21Ras scFv. Intravenous injection of CIK cells carrying KGHV500 *in vivo* significantly inhibits tumor growth, has little effect on normal organs, and is relatively safe.

## Introduction

Liver cancer is a common malignant tumor worldwide, with the fourth highest mortality rate 1. In China, the incidence of liver cancer is high, and liver cancer has the third highest mortality rate 2. The current treatment options for liver cancer include surgical resection, local ablation, liver transplantation, radiation therapy, transcatheter arterial chemoembolization, and systemic chemotherapy, but the 5-year survival rate of liver cancer patients remains extremely low 3. While multi-target kinase inhibitor sorafenib could prolong median survival of patients with advanced liver cancer, approximately 70% of patients develop resistance or serious adverse reactions to sorafenib 4-9.

Ras is a classic oncogene involved in cell proliferation, differentiation, and apoptosis 10. Ras mutations contribute to 30% of all human cancers 11. Among three Ras genes KRAS, NRAS, and HRAS, NRAS and HRAS mutations are more common in liver cancer 12. Furthermore, HRAS overexpression plays a significant role in the development of liver cancer 13. Therefore, Ras is a potential target for liver cancer therapy.

In previous study, we constructed p21-Ras single-chain variable fragment antibody (anti-p21Ras scFv) that could bind mutant p21Ras and overexpressed p21Ras (K-p21Ras, N-p21Ras, and H-p21Ras) 14. In addition, we utilized recombinant oncolytic adenovirus KGHV300 carrying anti-p21Ras scFv for intratumoral injection to inhibit tumor growth *in vivo* 15. However, intratumoral injection is only suitable for superficial tumors and is not useful for deep tumors. Intravenous injection of adenovirus *in vivo* therapy has the hidden danger of systemic virus infection 16. Therefore, it is particularly urgent to solve the problem of adenovirus delivery from veins to tumors. Cytokine-induced killer (CIK) cells can recognize tumors through related receptors and will not damage normal cells. In present study, we employed CIK cells to deliver KGHV500 into the tumor and investigated anti-tumor effects and safety *in vivo*.

## Materials and Methods

### Cell lines

Human liver cancer Huh7 cell line, normal liver LO-2 cell line, and HEK293 cells were obtained from Kunming Institute of Zoology, Chinese Academy of Sciences. Cells were cultured in DMEM supplemented with 10% heat-inactivated fetal bovine serum (FBS), 100 U/mL penicillin and 100 μg/mL streptomycin at 37°C with 5% CO_2_ atmosphere.

### Recombinant oncolytic adenovirus construction

Wild-type adenovirus (Ad5) was used to construct recombinant oncolytic adenoviruses KGHV400 and KGHV500. Ad5 cilia gene was replaced with Ad35 cilia gene to promote virus binding to CD46 receptor on CIK cells. In KGHV500, anti-p21Ras scFv gene was inserted to express anti-p21Ras scFv constitutively, while in KGHV400 there was no insert of anti-p21Ras scFv gene. HEK293 cells were used to amplify KGHV400 and KGHV500. Virus stocks were collected, and purified by CsCl density gradient centrifugation. Virus titer was determined by the TCID50 method. Huh7 and CIK cells were infected with viruses at a MOI of 100.

### Electron microscopy

The cells were fixed in 2% glutaraldehyde solution for 40 min, washed with PBS and fixed with 1% osmium acid for 30 min. Next the samples were dehydrated with ethanol and embedded in EPON 618 epoxy resin. The embedded samples were cut into 1-2 μm sections using an ultramicrotome, stained with uranyl acetate, and observed under electron microscope.

### Preparation of CIK cells

Peripheral blood mononuclear cells (PBMCs) were isolated from healthy volunteers by lymphocyte separation and density gradient centrifugation. PBMCs were cultured in RPMI 1640 medium containing 10% FBS and 1,000 U/mL IFN-γ (Peprotech, USA). The medium was replaced every three days, and 300 U/mL IL-2 was added. After 14 days, CIK cells were harvested at 90% confluency. CIK phenotypes were characterized by immunohistochemistry. Briefly, the cells were incubated with anti-CD3 mAb (ZSGB-Bio, ZA0503, China), anti-CD56 mAb (MXB, Kit-0028, China), and anti-CD46 mAb (Abcam, EPR4014, UK) overnight at 4 °C. After washing, the cells were incubated with horseradish peroxidase labeled goat anti-mouse IgG antibody (ZSGB-Bio, ZB5305, China) at 37 °C for 30 min, and then incubated with diaminobenzidine. Counterstaining was performed using hematoxylin, and the slides were sealed with neutral balsam.

### Flow cytometry

CIK cells were incubated with anti-CD16-APC (eBioscience), anti-CD56-PE (Biolegend), and anti-CD3-PerCP (Biolegend). Samples were centrifuged for 10 min at 1,500 rpm at room temperature, washed twice in PBS and subjected to flow cytometry on FC500 cytometer (Beckman-Coulter). The results were analyzed using Flow Jo software.

### MTT assay

Huh7 cells were plated at a density of 1×10^4^ cells per well in 96-well plates. After 1-3 d, cells were infected with KGHV500 at a MOI of 100. Then, 20 μL MTT agent (Amresco, USA) was added to each well on 1, 2, 3, 4, and 5 days after infection. After incubation for 4 h at 37 °C, MTT solution was removed and 100 μL dimethyl sulfoxide was added to each well, and the plates were shaken for 10 min. The optical density (OD) value of each well was measured in a microplate reader (Bio-Rad).

### Wound healing assay

Huh7 cells were infected with KGHV500 at a MOI of 100. After 24 h, the cells were seeded in 6-well plates and cultured to 90% confluency. A straight scratch was made in the cell monolayer with a 200 μL tip, and the cells were washed three times with PBS to remove detached cells. Cells were cultured for 48 h, and cell migration over the scratch was monitored with an inverted microscope. The migration distance was measured with ImageJ software.

### Transwell invasion assay

Transwell insert (Corning 3422 USA) was divided into upper and lower chambers by a microporous membrane. The upper chamber was coated with Matrigel. Huh7 cells were infected with KGHV500 at a MOI of 100. After 24 h, the cells were seeded in the upper chamber, and 500 μL of medium was added to the lower chamber. Cells were incubated in the Transwell insert for 24-48 h at 37 °C. The cells in the upper chamber were removed with cotton swab, and the membranes were fixed in methanol for 15 min and stained with Giemsa for 15 min. Cells that migrated through the Matrigel were counted under an inverted microscope, and five fields in each sample were counted.

### TUNEL assay

Cells or tumor sections were fixed and stained using TUNEL kit (Roche Diagnostics, Indianapolis, IN, USA) following the manufacturer's protocols. The nuclei were stained as blue by 4',6-diamidino-2-phenylindole.

### Animal xenograft model

Huh7 (1 × 10^7^/200 μL) cells were injected subcutaneously into the right axilla of BALB/c nude mice (Vital River Laboratories, Beijing, China). When the tumors grew to average diameter of 5 mm, the animals were randomized into 5 groups and received intravenous injection of either PBS, CIK cells, KGHV500, CIK + KGHV500, or CIK + KGHV400, respectively. Tumor size was measured with Vernier caliper every three days. Tumor volume was calculated according to the formula: V= (length × width^2^) ÷ 2 16. When tumors in PBS group became ulcerated, mice were sacrificed. The dissected tumors were embedded with paraffin for immunohistochemical analysis. The anti-FLAG tag antibody (Abnova, MAB9744, China) and anti-human adenovirus hexon mouse mAb (Novus, NBP211638, USA) were used to detect KGHV500 adenovirus.

### Real-time PCR

Total RNA was extracted from tumor tissues using the ReliaPrep^TM^ Total RNA extraction kit (Promega, USA). RNA was reverse transcribed into cDNA using the GoScript^TM^ Reverse Transcription system (Promega, USA). PCR was performed with GoTaq qPCR Master Mix (Promega, USA). The primers were as follows: survivin CCACTGAGAACGAGCCAGAC and CGCACTTTCTCCGCAGTTTC; p53 CCTGAGGTTGGCTCTGACTG and CTTCTTTGGCTGGGGAGAGG; caspase-7 AAGCTGACTTCCTCTTCGCC and TCCAGGTCTTTTCCGTGCTC; caspase-3 AAATACCAGTGGAGGCCGAC and AACCCGGGTAAGAATGTGCA; bcl-2 GGGGTCATGTGTGTGGAGAG and ACCTACCCAGCCTCCGTTAT; GAPDH TGACAACAGCCTCAAGAT and GAGTCCTTCCACGATACC. The reaction conditions were as follows: 94 °C for 15 s, 60 °C for 30 s, and 72 °C for 30 s (40 cycles). The data were analyzed using Bio-Rad CFX96 Manager software.

### Western blotting

Mouse tissues were lysed in RIPA buffer on the ice for 30 min. The lysates were centrifuged and the supernatants were collected for SDS-PAGE gel electrophoresis. The proteins were then transferred to polyvinylidene fluoride membranes, which were incubated with antibodies for survivin, p53, Caspase-7, Caspase-3, Bcl-2, and β-actin (all from Abcam) overnight. Next, the membranes were incubated with horseradish peroxidase labeled goat anti-mouse IgG antibody (ZSGB-BIO, Beijing, China), and developed with enhanced chemiluminescence kit (Pierce).

### Statistical analysis

All data were expressed as mean ± standard deviation and analyzed using SPSS 22.0 software (SPSS, Chicago, USA). Differences in multiple groups were analyzed by one-way analysis of variance (ANOVA). Statistical significance was indicated by P-value <0.05.

## Results

### Infection of Huh7 cells with recombinant oncolytic adenovirus KGHV500

**Figure 1:**
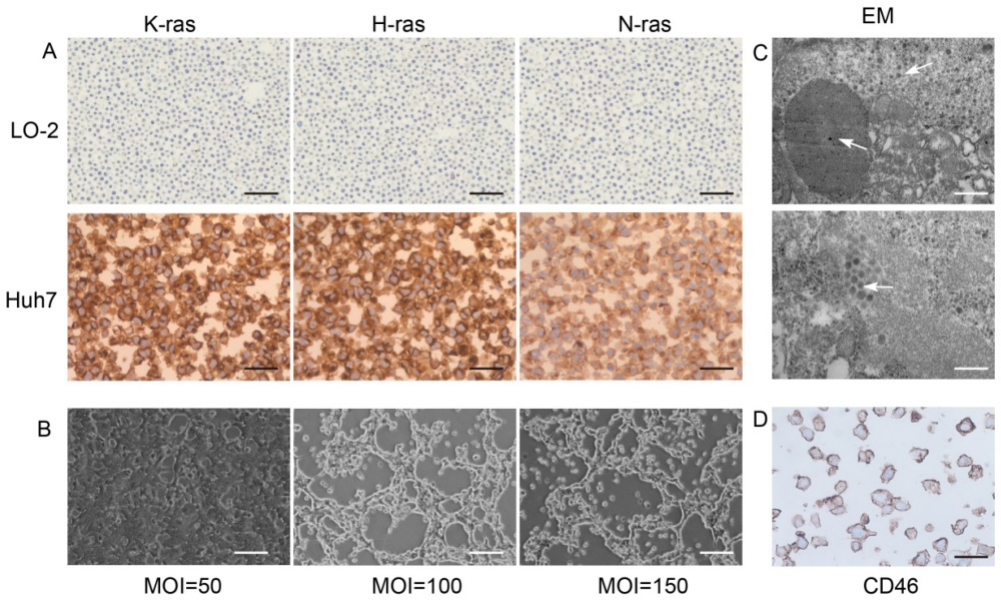
**Characterization of KGHV500-infected Huh7 cells.** (A) K-Ras, H-Ras, and N-Ras were stained strong in Huh7 liver cancer cells but not in LO-2 normal liver cells. Bar: 100 µm. (B) After 48 h of infection with KGHV500 (MOI = 100), the cytopathic effect (CPE) was the weakest. Bar: 200 µm. (C) Virus particles were observed in the cytoplasm and the nuclei of Huh7 cells were observed by electron microscopy (marked by arrows). Bar: 1 µm or 500 nm. (D) CD46 on the surface of Huh7 cells was detected by IHC. Bar: 100 µm.

Ras proteins p21K-Ras, p21H-Ras, and p21N-Ras were overexpressed in Huh7 cells but not in normal LO-2 hepatocytes (Figure 1A). Huh7 and KGHV500 were co-cultured, and the optimal ratio of virus-infected cells was determined by monitoring the cytopathic effect (CPE). After 48-h infection, at a MOI of 100, CPE was weak, and fluorescence increased (Figure 1B). In addition, numerous virus particles were present in the cytoplasm and nuclei of Huh7 cells infected with oncolytic adenovirus (Figure 1C). CD46 was expressed on Huh7 cell membrane and bound to receptor of KGHV500 (Figure 1D). These results validated that Huh7 cells were successfully infected with KGHV500.

### KGHV500 inhibited liver cancer cell migration

**Figure 2:**
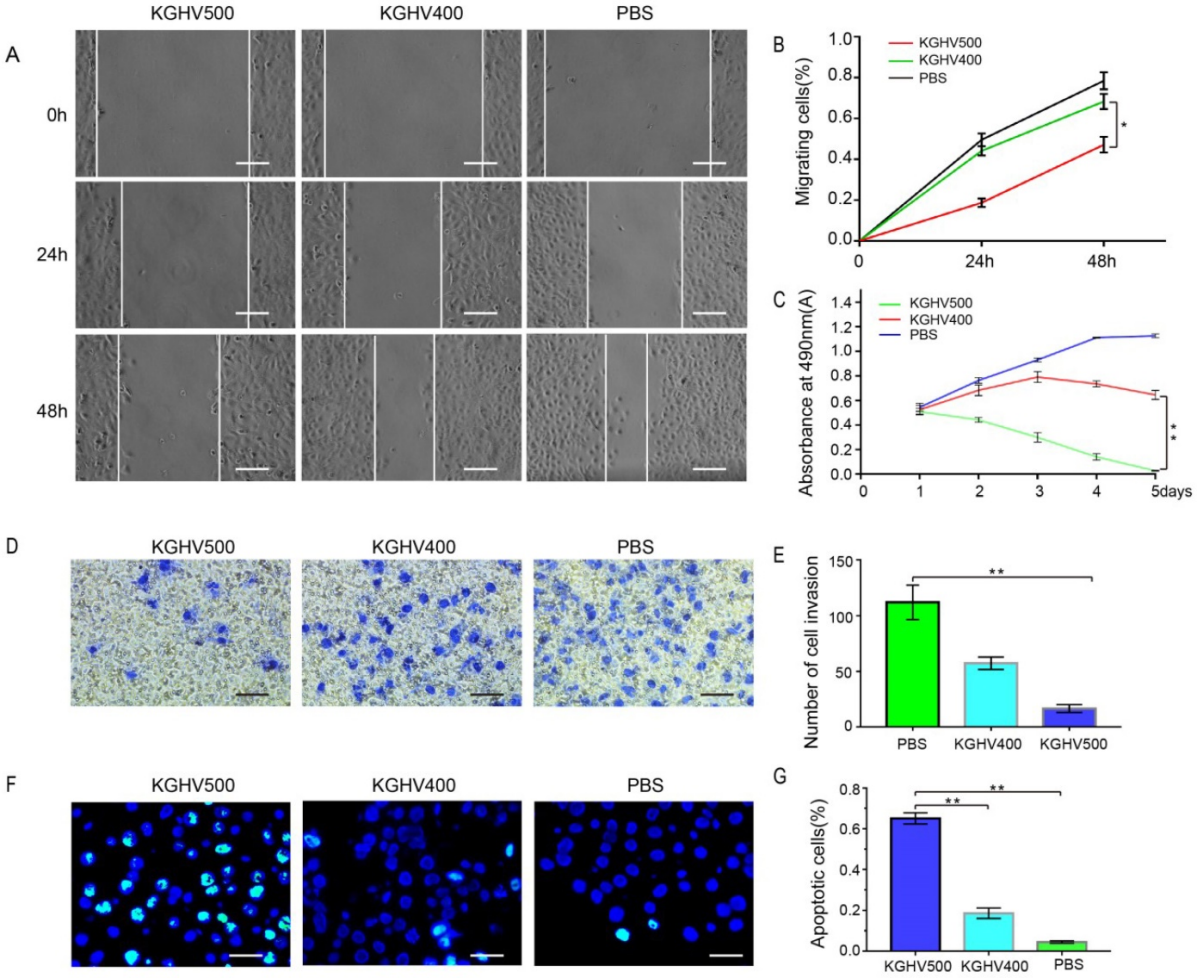
***In vitro* anti-tumor effects of KGHV500.** (A and B) In wound healing assay, healing in KGHV500 group was slower than in KGHV400 or PBS group, and the number of cells that migrated was less than the other groups (P < 0.05) Bar: 50 µm. (C) In MTT assay, KGHV500 was more toxic to the cells than KGHV400 or PBS (P < 0.01). (D and E) In Transwell assay, KGHV500-infected Huh7 cells were significantly less invasive than the other groups (P < 0.01). Bar: 40 µm (F and G) In TUNEL assay, the number of apoptotic Huh7 cells in KGHV500 group was higher than that of other groups (P < 0.01). Bar: 100 µm *P < 0.05, **P < 0.01.

The wound healing assay demonstrated that the number of migrated cells in KGHV500-treated group was less than that of control group (Figure 2A). The percentage of migrated cells was 18.68 ± 2.11% at 24 h and 47.14 ± 3.86% at 48 h after KGHV500 infection; the percentage of migrated cells was 44.12 ± 2.29% at 24 h and 68.29 ± 3.70% at 48 h after KGHV400 infection. In Huh7 control group, the percentage of migrated cells was 49.55 ± 3.10% at 24 h and 78.49 ± 4.23% at 48 h (Figure 2B).

### KGHV500 inhibited liver cancer cell growth

MTT assay showed that cell viability of KGHV500-treated group decreased over time, while cell viability of KGHV400-treated group increased initially and decreased slowly, indicating that KGHV500 inhibited Huh7 cell growth (Figure 2C).

### KGHV500 inhibited liver cancer cell invasion

Transwell assay demonstrated that the number of invading cells in KGHV500-treated group was less than that in other groups (Figure 2D). The number of invading cells in KGHV500 group was 16.20 ± 3.19, compared to 57.40 ± 5.00 in KGHV400 group and 112.00 ± 13.83 in control group (Figure 2E).

### KGHV500 induced apoptosis in liver cancer cells

TUNEL assay showed that KGHV500 treatment increased the number of apoptotic cells compared to the other groups (Figure 2F). The percentage of apoptotic cells was 65.08 ± 2.72% in KGHV500-treated group, 18.59 ± 2.59% in KGHV400-treated group, and 4.43 ± 0.59% in control group (Figure 2G).

### KGHV500 bound to CIK cells

**Figure 3:**
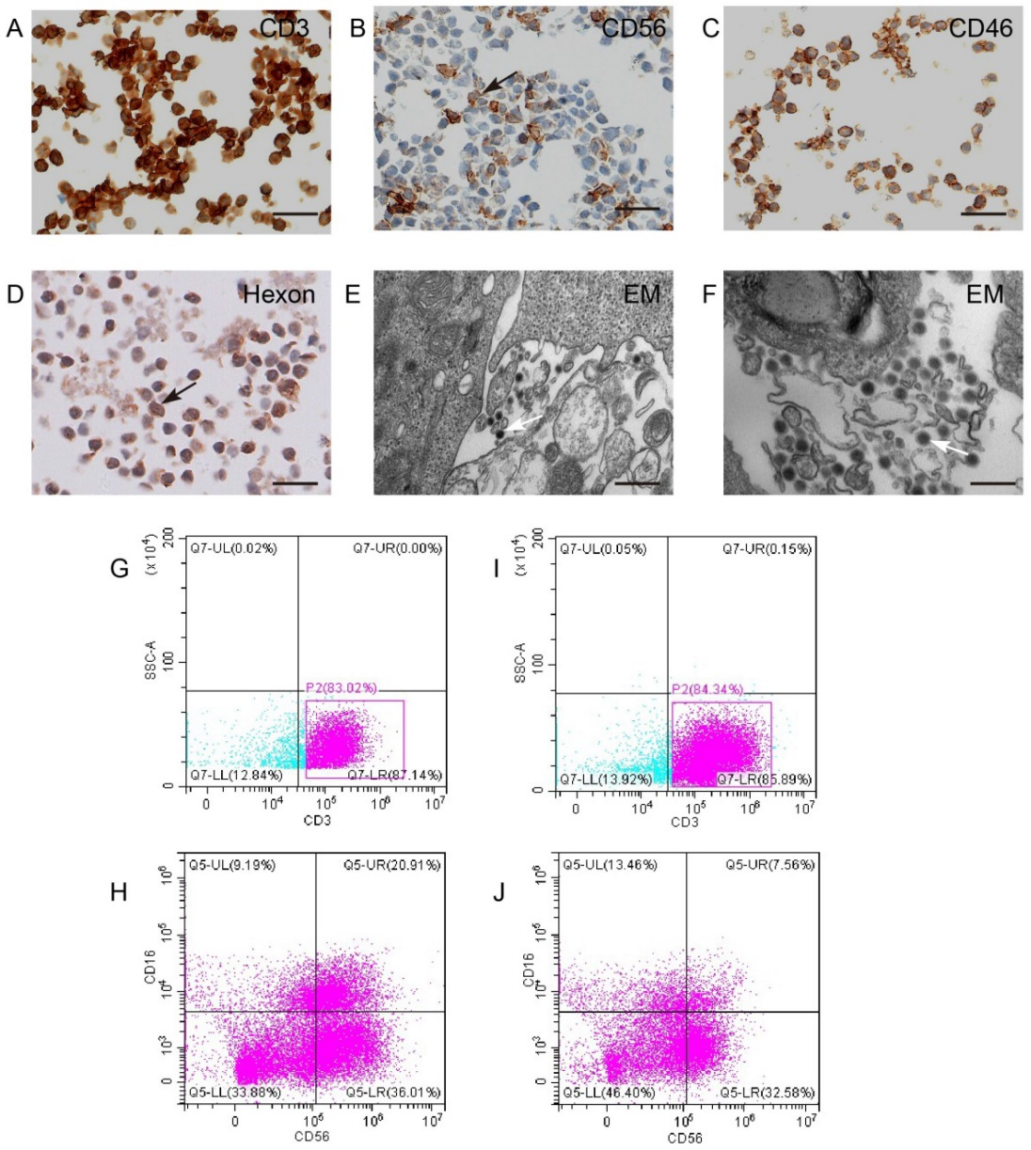
**CIK cells could carry KGHV500.** (A and B) CIK cell markers CD3 and CD56 were detected by IHC. Bar: 100 µm. (C) CD46 on the surface of CIK cells was detected by IHC. Bar: 100 µm. (D) The adenovirus hexon was detected in CIK cells by IHC. Bar: 100 µm. Strong staining was marked by arrows. (E and F) Electron microscopy showed that many virus particles accumulated on CIK cell membrane (marked by arrows). Bar: 500 nm. (G-J) Flow cytometry of CD3 and CD56 expression of CIK cells before and after KGHV500 infection.

CIK cells were identified by surface specific markers CD3 and CD56 (Figure 3A,B). To test whether KGHV500 could infect CIK cells, immunohistochemistry was used to detect CD46 (Figure 3C) and adenovirus hexon (Figure 3D) on CIK cell surface. Electron microscopy confirmed the presence of KGHV500 particles on the surface of CIK cells (Figure 3E,F). In addition, we performed flow cytometry to detect the changes in the phenotypes of CIK cells before and after KGHV500 infection (Figure 3G-J), and found that CD3 positive cells before and after infection were greater than 80%, while CD3^+^CD16^+^CD56^+^ cells decreased from 20.91% before infection to 7.56% after infection. These results indicated that CIK cells were successfully infected and could bind recombinant adenovirus KGHV500.

### *In vivo* anti-tumor efficacy of CIK cells carrying KGHV500

**Figure 4:**
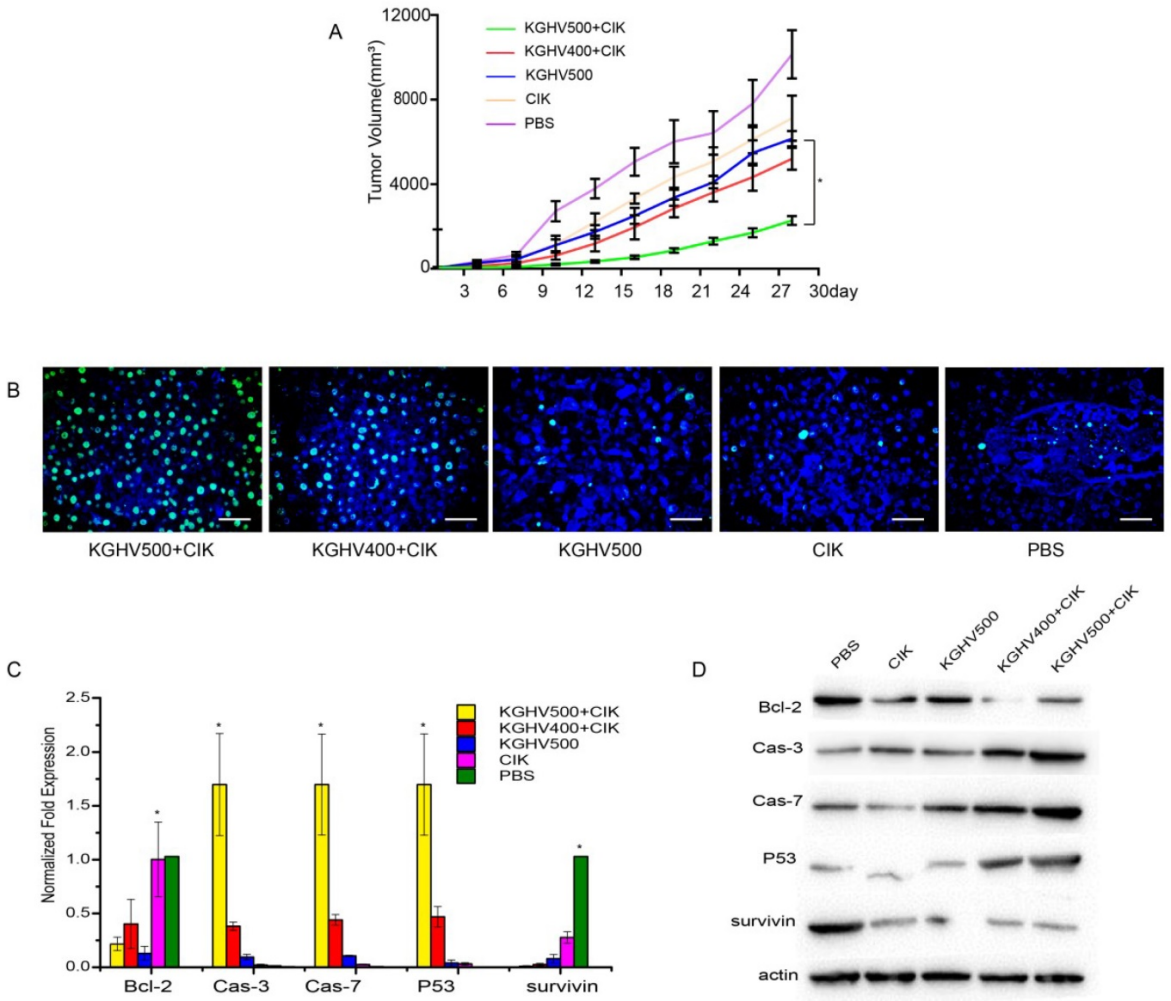
** Anti-tumor efficacy of CIK cells carrying KGHV500 *in vivo*.** (A) Tumor growth curve showed that CIK + KGHV500 tumor size was significantly smaller compared to other groups. (B) TUNEL assay of apoptotic tumor cells in each group. Bar: 100 µm. (C) The mRNA expression of apoptosis-related genes in xenografts was detected by PCR. *P < 0.05. (D) Western blot analysis of apoptosis-related proteins in xenografts.

Tumor volume of KGHV500 + CIK cells treated mice was the smallest, indicating that KGHV500 + CIK cells significantly inhibited the growth of mouse xenograft tumors (Figure 4A). TUNEL assay indicated that the apoptotic rate of KGHV500 + CIK group was 81.50 ± 1.72%, higher than that of KGHV400 + CIK group (55.53 ± 2.82%), KGHV500 group (19.47 ± 2.29%), CIK cell group (11.33 ± 0.93%), and PBS group (7.27 ± 0.77%) (Figure 4B). In addition, we detected the expression of apoptosis-related genes in the xenograft tissue by PCR and Western blot analysis (Figure 4C,D). KGHV500 + CIK treatment decreased the expression of anti-apoptotic proteins *Bcl-2* and survivin and increased the expression of pro-apoptotic proteins p53, caspase-3, and caspase-7. These data indicated that KGHV500 + CIK cells induced apoptosis in Huh7 liver cancer cells and inhibited tumor growth in nude mice.

### Safety and distribution of KGHV500 + CIK cells *in vivo*

Next we performed immunohistochemistry and Western blotting to detect the distribution of adenovirus hexon and anti-p21Ras scFv in tumor and normal tissues and determine the safety of KGHV500 + CIK cells.

**Figure 5:**
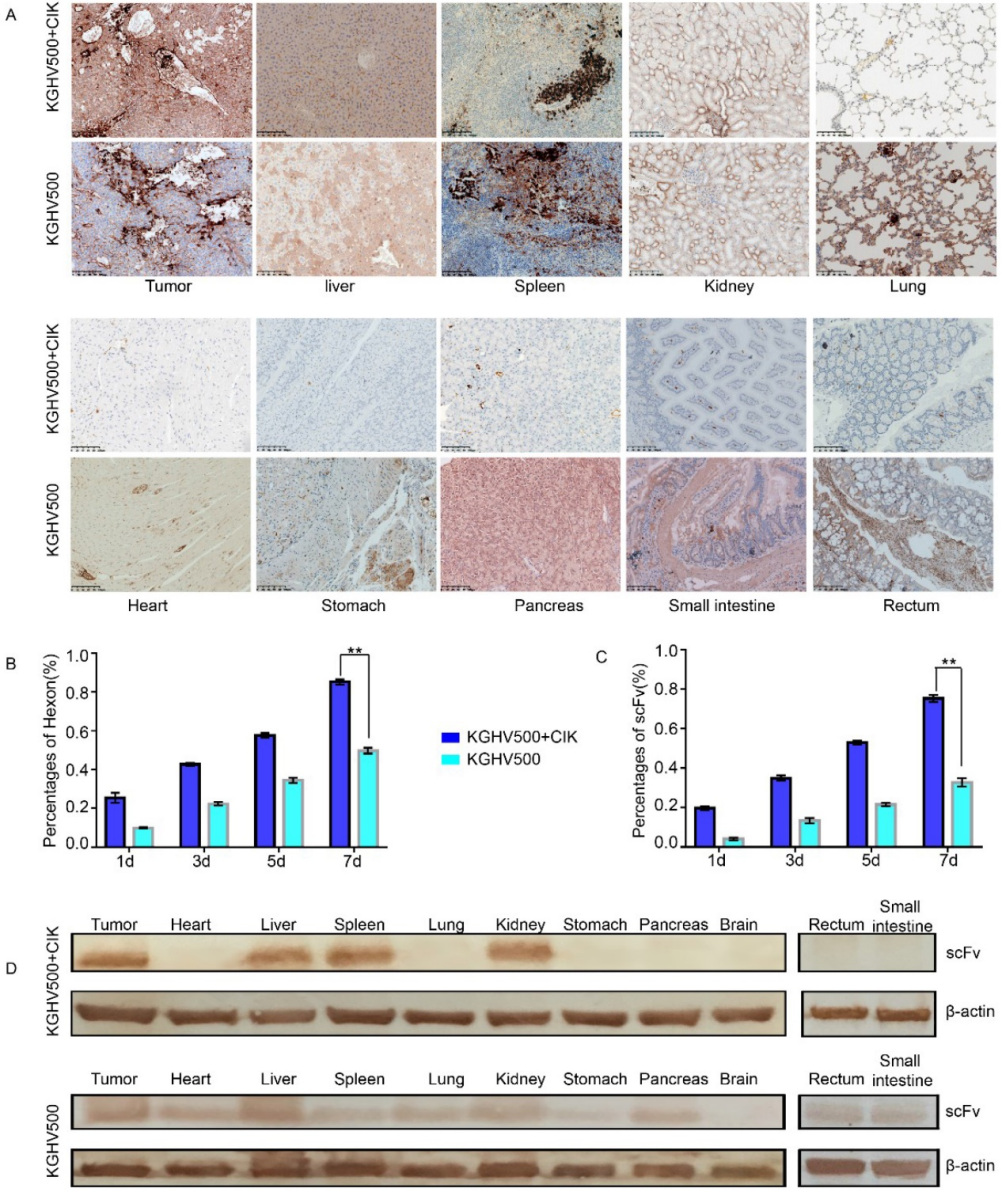
** Targeting and safety of CIK + KGHV500 cells *in vivo*.** (A) Expression of adenovirus hexon was detected by IHC. Bar: 100 μm. (B) In CIK + KGHV500 group, the percentage of tumors positive for adenovirus hexon was higher than that in KGHV500 group. **P < 0.01. (C) CIK + KGHV500 group had more anti-p21Ras scFv-positive cells in xenograft tumors than the KGHV500 group. **P < 0.01. (D) Western blot analysis of anti-p21Ras scFv expression in xenograft tumors and organs of nude mice.

In KGHV500 + CIK treatment group, adenovirus hexon was expressed in tumor tissue, the spleen, liver, and kidney, but not in the lung, heart, stomach, pancreas, large intestine, small intestine, or brain tissue. In KGHV500 treatment group, adenovirus hexon was detected in both tumors and normal tissue, except for brain tissue (Figure 5A). The percentages of adenovirus hexon positive cells in tumors from CIK + KGHV500 group were 25.45 ± 2.48%, 42.67 ± 0.72%, 57.56 ±1.23%, and 85.22 ± 1.40% on days 1, 3, 5, and 7 after administration, respectively. In KGHV500 group, the percentages were 9.89 ± 0.42%, 22.33 ± 0.98%, 34.44% ± 1.40%, and 49.78 ± 1.50% on days 1, 3, 5, and 7 after administration, respectively (Figure 5B). The percentage of adenovirus hexon positive cells in xenograft tumors was higher in CIK + KGHV500 group than in KGHV500 group. These results indicate that CIK cells carrying recombinant adenovirus have tumor targeting abilities and can deliver recombinant adenovirus to tumor site.

Immunohistochemistry demonstrated that the percentages of anti-p21Ras scFv positive cells in xenograft tumors from CIK + KGHV500 group were 19.67 ± 0.98%, 35 ± 1.36%, 53 ± 0.98%, and 75.33 ± 1.79% on days 1, 3, 5, and 7 after administration, respectively. In KGHV500 group, the percentages were 4.11 ± 0.69%, 13.44 ± 1.38%, 21.55 ± 0.88%, and 32.78 ± 2.08%, on days 1, 3, 5, and 7 after administration, respectively (Figure 5C). Therefore, carrying KGHV500 in CIK cells increased the expression of anti-p21Ras scFv in xenograft tumors.

Western blotting confirmed that in KGHV500 + CIK group, anti-p21 Ras scFv was expressed in tumor tissue, the liver, spleen, and kidney, while in KGHV500 group, anti-p21 Ras scFv was expressed in tumor tissue and all normal tissues, except for brain tissue (Figure 5D). Expression of anti-p21Ras scFv was consistent with the distribution of adenovirus hexon, indicating that CIK cells delivered KGHV500 to tumor tissues selectively to express anti-p21 Ras scFv.

## Discussion

RAS plays an oncogenic role in cancer development 10-13. Therefore, Ras inhibition is a potential strategy to treat liver cancer. The anti-p21Ras scFv constructed in our laboratory can block Ras signaling and target tumors with high expression of Ras 14, 15. However, there are hurdles to the application of anti-p21Ras scFv in cancer treatment, including efficient delivery to tumor cells, intracellular p21Ras protein binding, and achievement of sustained expression.

Oncolytic adenovirus continuously infects tumor tissues, allowing for efficient infection and maintenance of long-term gene expression 17. Moreover, it does not replicate in normal tissues, but replicates in cancer cells to kill cancer cells selectively 18. Therefore, we constructed recombinant oncolytic adenovirus KGHV500 that could specifically replicated in tumor cells and expressed anti-p21Ras scFv. *In vitro* experiments showed that KGHV500 successfully infected liver cancer cells, expressed anti-p21Ras scFv, inhibited liver cancer cell proliferation, migration, invasion, and induced apoptosis of liver cancer cells.

Although oncolytic adenovirus-mediated gene therapy has many advantages by intratumoral delivery, the delivery *in vivo* is a limiting factor since it could infect normal cells. For intravenous injection of virus, the virus is non-specifically adsorbed by vascular endothelial cells and blood cells, or neutralized by antibodies 19. In order to transport KGHV500 to tumor tissues and enhance the targeting and safety *in vivo*, we used CIK cells to carry KGHV500 to tumor tissues.

By immunohistochemistry and flow cytometry, we confirmed that CIK cells we induced had high expression of CD3 and CD56. CIK cells specifically recognize tumors without toxic effects on normal cells. CIK cells recognize tumors mainly by chemokine ligand (CXCL) 9, CXCL10, and CXCL11, or by binding of CIK cell surface NKG2D receptor to tumor cell surface MHC class I chain-related protein A or B ligand 20,21. Clinical studies have shown that CIK cell therapy alone is less responsive to patients with high tumor burden 22. Therefore, combining CIK cell therapy with oncolytic adenovirus treatment may be a better approach for cancer therapy.

Furthermore, we found KGHV500 distribution and anti-p21Ras scFv expression in the liver, spleen and kidney of nude mice. Adenovirus accumulation in the liver is associated with hepatotoxicity 23. In addition, 90% of intravenous adenoviruses are excreted by the liver 24. Adenovirus accumulation has been detected in the spleen, which may be related to the homing of peripheral lymphocytes 25. Detection of recombinant adenovirus in the kidney may be related to kidney metabolism 26. Our results indicate that CIK cells carrying KGHV500 significantly inhibited tumor growth *in vivo*, and were safe with little effect on normal tissues. In addition, our previous studies showed that CIK carrying KGHV500 inhibited the growth of lung cancer, colon cancer and gastric cancer in nude mice, indicating broad-spectrum anti-tumor efficacy of CIK carrying KGHV500 27-29.

In conclusion, we develop a novel strategy for the treatment of Ras-driven liver cancer by combining CIK cells with oncolytic adenovirus expressing anti-p21Ras scFv. Further preclinical studies are necessary to validate the potential of our strategy for the treatment of various cancers.
